# Fabry Disease: A Atypical Presentation

**DOI:** 10.7759/cureus.18708

**Published:** 2021-10-12

**Authors:** Cláudia Ferreira Tátá, Margarida Massas, Filipa Pinto, Nuno Caçador, Ana Luisa Silva

**Affiliations:** 1 Internal Medicine, Hospital do Espírito Santo de Évora, Évora, PRT; 2 Internal Medicine, Hospital do Espirito Santo de Évora, Évora, PRT; 3 Radiology • Neuroradiology, Hospital do Espirito Santo de Évora, Évora, PRT

**Keywords:** nervous central system, enzyme alpha-galactosity a, gla gene, lysosomal storage disease, neuropathic pain, white matter lesions, fabry disease

## Abstract

Fabry Disease (FD) is a rare X-linked recessive disease caused by mutations in the GLA gene that lead to a decrease or lack of activity of the enzyme alpha galactosyl A. This lysosomal storage disorder results in progressive damage and dysfunction of several organs and, depending on the type of mutation and gender of the patient, and it may have different manifestations. As FD is a multisystem disease with a progressive character and varying severity, the diagnosis can be challenging, especially when it comes to non-classical forms. As this is a hereditary disease, its diagnosis impacts not only the patient but also his family, making an accurate and timely diagnosis even more important.

We present the case of a 59-years-old man diagnosed with non-classical FD, with previous neurological and psychiatric complaints, who was admitted to the Emergency Department (ED) with a generalized tonic-clonic seizure that required orotracheal intubation for airway protection and transferred to an Intensive Care Unit (ICU).

## Introduction

Fabry Disease (FD) is a rare inherited lysosomal storage disease with an X-linked inheritance pattern. It results from decreased or absent lysosomal enzyme α-Galactosidase A (α-Gal A) activity, caused by mutations of the GLA gene, the gene responsible for encoding it [1-3]. In turn, the decrease or absence of α-Gal A activity results in the progressive accumulation of globotriaosylceramide (Gb3) and related glycosphingolipids in lysosomes, causing progressive damage and dysfunction of several organs, namely the cardiovascular, renal and nervous systems [3].

The manifestations and severity of the disease depend not only on the patient's gender but also on the type of mutation in the GLA gene, with more than a thousand variants of the gene described, many of which still have no well-established pathological significance [4]. Given the variability of disease manifestations and progression, diagnosis can become a challenge. Early diagnosis allows for earlier initiation of enzyme replacement therapy and thus delaying disease progression, so FD should be a diagnostic hypothesis to consider, particularly in patients with stroke/cerebrovascular disease, chronic kidney disease or hypertrophic cardiomyopathy [3].

Regarding the central nervous system, the accumulation of Gb3 and its derivatives occurs mainly in endothelial cells and vascular smooth muscle, being also responsible for glial deposition and neuron damage. In the peripheral nervous system, these glycolipids accumulate in the sympathetic ganglia and endothelial cells of the blood vessels that supply the nerve fibres [1]. Thus, the neurological manifestations of FD can vary in severity and include acute or chronic cerebrovascular events in any of the vascular territories, neuropathic pain, cochleovestibular dysfunction and cognitive deficit (from mild to severe) as well as psychiatric symptoms [1].

The p.A143T mutation is considered a variant of uncertain meaning. Its role in FD is somewhat controversial, with cases described with a phenotype that varies from classic FD to an asymptomatic carrier with normal α-Gal A activity [5]. However, there seems to be an association of this mutation with late-onset disease. The disease phenotype caused by this mutation involves only one of the main organ systems affected by FD [6,7], particularly the central [7-9] and peripheral nervous systems [10]. The p.A143T mutation and its pathological significance must be evaluated case-by-case basis [5].

## Case presentation

We present the case of a 59 years-old self-employed male with a 10-year history of high blood pressure (HBP), medicated with two anti-hypertensive drugs with irregular compliance and poor blood pressure control. Significantly, his mother started presenting signs and symptoms of dementia early onset (50 years old) and died from chronic depression and cognitive deterioration, with repeated hospital admissions at the Psychiatric ward. Furthermore, he had one brother diagnosed with schizophrenia and two other brothers with a history of alcohol abuse. He also had two daughters from which one suffered from severe depression.

This patient was referred to a history of psychomotor slowing, sporadic episodes of dysphagia, irritability, emotional lability, anxiety, decreased hearing, vertigo and paraesthesia, and pain in both lower limbs for the past year. He was taken to the ED due to an episode compatible with a generalized tonic-clonic seizure disorder and sphincter incontinence (faecal and urinary). Upon admission to the ED, he presented an altered level of consciousness, with 9 points on the Glasgow Coma Scale (GCS), conjugated deviation of the gaze to the right, flaccid plegia of the four limbs, and indifferent right plantar skin reflex, which was normal at the left foot and HBP. The medical team interpreted his condition in the context of the post-critical period. From the complementary study, the arterial blood gases showed moderate mixed acidaemia (pH 7.13, pCO_2_ 83.3mmHg, HCO_3_-28.0mmol/L), respiratory predominance, hyperlacticaemia (lactate 6.2mmol/L) and hypokalaemia. Cranial computed tomography (CT) with angiography showed cerebral atrophy inconsistent with the patient's age and leukoaraiosis, not documenting acute ischemic or haemorrhagic lesions or presence of thrombi or other changes in cerebral arterial vascularization.

During his stay at the ED, the patient suffered a second convulsive seizure, which was controlled with diazepam. He was started on levetiracetam 1g. Despite an initial improvement in consciousness, the patient progressed to a third seizure, with maintained GCS below 8. He was then submitted to orotracheal intubation and mechanical ventilation for airway protection, under continuous sedation with propofol and midazolam. Lumbar puncture was performed, with a cytochemical and bacteriological unchanged cerebrospinal fluid. This was tested for neurotropic viruses without relevant findings. He was transferred to the Intensive Care Unit (ICU).

During his stay at the ICU, he had no recurrence of convulsive crises when weaning from sedation, having completed ventilatory weaning and subsequent extubating uneventfully. The patient remained hemodynamically stable, though he needed three anti-hypertensive drugs to control his HBP.

He was transferred to the Medicine ward, maintaining clinical stability, with a slight slowing of speech and slight distal atrophy of the upper limbs, despite maintaining muscle strength. He was submitted to a neuropsychological assessment that revealed articulatory effort with anomalous pauses and slowed recall, with difficulty in executive tasks, as well as a decrease in the speed of information processing.

**Figure 1:**
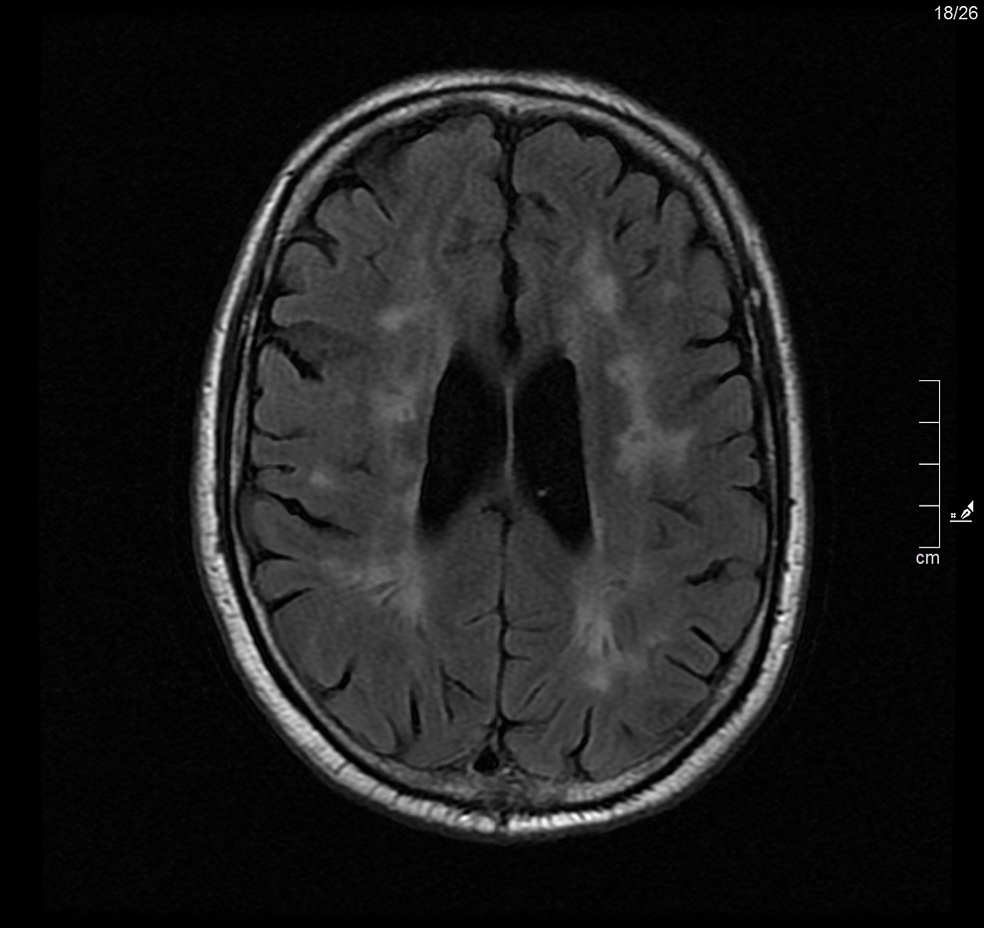
MRI of the brain - FLAIR T2 sequence - diffuse changes in the periventricular and deep subcortical white matter Change in signal intensity with hyperintense areas and lesions in FLAIR T2 sequence located in periventricular white matter, radiated crowns and subcortical white matter of both cerebral hemispheres.

**Figure 2:**
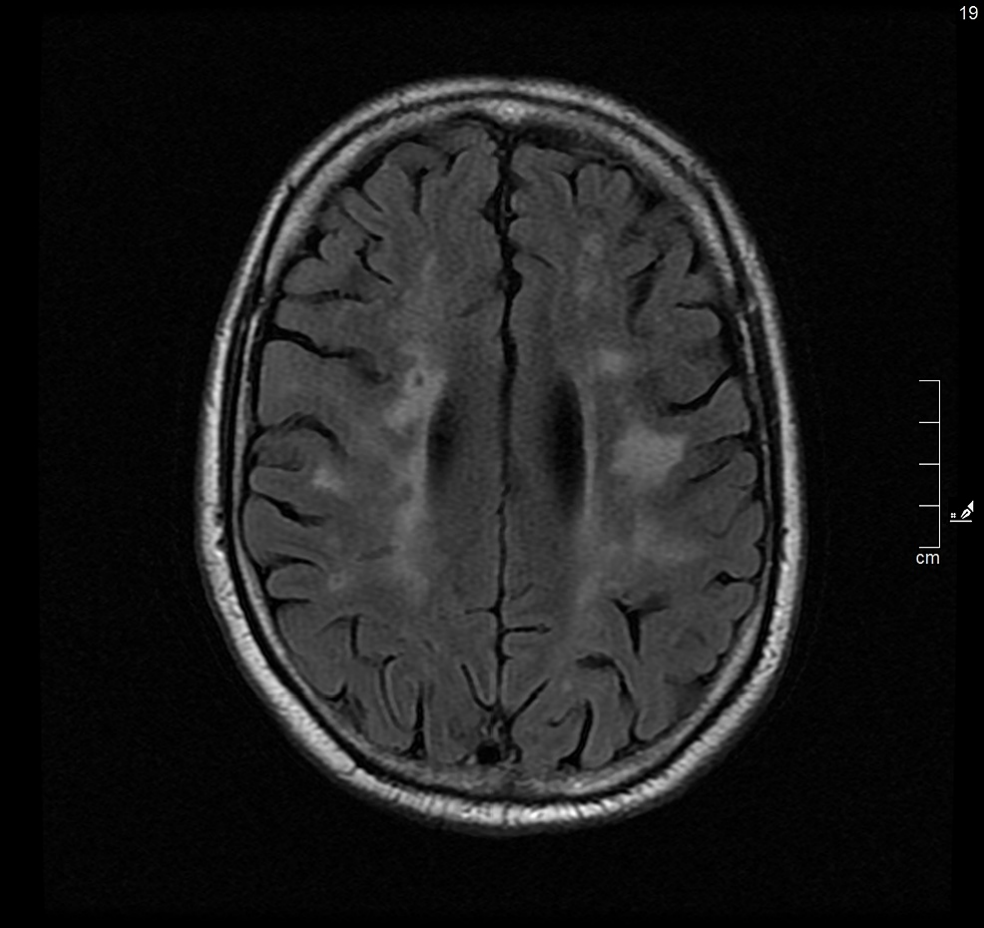
MRI of the brain - FLAIR T2 sequence - diffuse changes in the periventricular and deep subcortical white matter Change in signal intensity with hyperintense areas and lesions in FLAIR T2 sequence located in periventricular white matter, semivowel centers and subcortical white matter of both cerebral hemispheres.

Magnetic resonance imaging (MRI) of the brain was performed, which identified in the T2 FLAIR sequences (Figure 1, 2) multiple areas of hyperintensity periventricular and subcortical frontoparietal white matter without restriction to diffusion, coexisting with other lacunar nucleus-capsular and bilateral thalamic sequelae lesions.

The Electroencephalogram (EEG) showed electrogenesis of a well-structured base overlaid with left temporal slowing without apparent paroxysms indicating left temporal regional dysfunction, not detecting associated epileptiform activity in this study; Transcranial Doppler echo documented increased pulsatility indices translating arterial inelasticity; Doppler echocardiography of the neck vessels showed diffuse carotid atheromatous without hemodynamic repercussions; Transthoracic echocardiogram and 24-hour Holter did not show relevant changes. In the analytical study, hypercholesterolemia and hypertriglyceridemia were highlighted. The prothrombotic study and the study of reversible causes of dementia did not reveal any changes.

Due to the difficulty to control hypertension, the study of causes of secondary HBP was started, and the main causes were excluded. The renal echography did not show renal artery stenosis or renal parenchyma alterations. Given the history of cognitive decline with mood alterations with one year of evolution and the alterations described in the MRI, the diagnosis of epilepsy of vascular etiology and important ischemic cerebrovascular disease (CVD) was admitted. However, the MRI changes are excessive for the patient's age, and possible genetic causes were investigated, namely the search for genes NOTCH3 (CADASIL), HTRA1 and Mitochondrial Encephalopathy Lactic Acidosis Stroke-like episodes (MELAS), which were excluded, and FD. 

During admission, antiepileptic therapy was progressively adjusted without recurrent seizures. He repeated EEG, which did not document epileptiform activity. He remained neurologically stable, without focal neurological deficits, calm and oriented in time, space, and person, although with slowed speech. He was discharged and continued the etiological investigation at the Internal Medicine and Neurology outpatient clinics.

The study of determining the enzymatic activity of alpha-galactosidase A in dried blood on filter paper revealed a partial deficiency (5.25 pmol/h/puncture, reference levels 8.75 - 15.6 pmol/h/puncture) not diagnostic for FD. Thus, a sample was sent to confirm the result in plasma and total leukocytes. This test revealed an enzyme deficiency of alpha-galactosidase A in leukocytes (6.9 nmol/h/mg protein, reference levels 36 - 80 nmol/h/mg protein) and a non-significant increase in globotriaosylsphingosine (2, 1 nmol/L reference levels 0 - 1,3 nmol/L). The presence, in hemizygosity, of the genetic variant c.427G>A (p.A143T) was also identified.

A diagnosis of non-classical FD was admitted, and a study of other manifestations of FD and family studies and genetic counselling was initiated and a study of the manifestations in other organs.

## Discussion

FD was identified by Anderson and Fabry in 1998 and is characterised by deficient α-Gal A activity [11]. In its classic form, the first, more nonspecific, disease symptoms occur in childhood or adolescence [2]. Neurological, cardiovascular, and renal complications usually appear in the third or fourth decade of life, with the main clinical manifestations of FD being skin lesions (angiokeratomas), peripheral neuropathic pain (acroparesthesia), renal failure, cardiomyopathy and cerebrovascular disease [3]. In its non-classical form, FD is a disease of variable involvement and severity, becoming a diagnostic challenge, with a mean delay in diagnosis of 13.7 and 16.3 years in men and women, respectively [4].

In this case report, FD presented with psychiatric and neurological symptoms with about a year of evolution, culminating in convulsive crises that motivated the visit to the ED. White matter lesions (WML), observable on MRI, are a common incidental finding in patients over 65 years of age but are rare in patients under 50 years of age [12]. The number of lesions is associated with neurological and psychiatric disorders, such as cognitive deficit and decline, depression, or epilepsy [13], with age and essential arterial hypertension being the main risk factors, alongside other cardiovascular risk factors [14]. Cerebral WML is common in FD, detectable in the MRI in 42.81% of patients, without preferentially affecting a gender or specific location [15,16].

The case presented is interesting both because of the presentation with epilepsy and the patient's age. Although he is not a patient under 50 years old, due to cognitive dysfunction, neuropathic pain, subjective hearing loss that raises the hypothesis of cochleovestibular dysfunction and psychiatric symptoms, together with lesions compatible with small vessel microangiopathy, a common expression of cerebral vasculopathy in FD [17], it was decided to continue the study of less frequent causes of WML, despite these could be justified by the patient risk factors (hypertension, smoking and dyslipidaemia).

Thus, FD screening was carried out, consisting of determining the enzymatic activity of alpha-galactosidase A in dried blood on filter paper. Although this test was not conclusive, the enzymatic activity of alpha-galactosidase A in leukocytes was determined, and the result was compatible with FD diagnosis. Hemizygosity for the p.A143T mutation was also identified. This mutation has an association with FD considered controversial, and some authors consider it non-pathogenic or "unlikely to be associated" with FD [5,7], as well as authors who propose an association with late-onset and oligosymptomatic forms of FD [5,6,7,10,18,19]. Some authors are more specific, referring to an association with preferentially affecting neurological disease [5,8,10] and preferentially affecting cardiovascular disease [9]. Thus, it seems reasonable to admit that the approach should be individualised for patients with this mutation. Regarding the case presented, which carries the p.A143T mutation (thus considered a variant of unknown significance), given that there is clinical and biochemical evidence of the disease (reduced leukocyte enzymatic activity), FD is considered as the final diagnosis.

After this diagnosis, it is necessary to look for other manifestations of FD, namely renal, cardiovascular (through cardiac MRI), ophthalmological (cornea verticillata and Fabry cataract). In the case of this patient, he was referred to these specialities for further study. Family study and genetic counselling is also essential, as well as referral to a specialist, since, as the patient has WML, he will be a candidate to initiate targeted therapy.

## Conclusions

The case presented is rare given the unusual presentation (convulsive crises) of an already rare disease. Despite the patient's history of arterial hypertension, the diagnosis of non-classical FD has to be considered due to the history of previous neurological symptoms (cognitive dysfunction, neuropathic pain, subjective hearing loss that raises the hypothesis of cochleovestibular dysfunction) and psychiatric symptoms, together with small vessel microangiopathy with a high load of lesions given the patient's age. As it is a hereditary disease, its diagnosis impacts the patient and his family. Thus, being alert to FD's manifestations, even if subtle, diagnosing FD and starting treatment when indicated is essential to change the natural history of the disease and the quality of life of a patient and their family.
